# Comparative Genomics and Drug Resistance of a Geographic Variant of ST239 Methicillin-Resistant *Staphylococcus aureus* Emerged in Russia

**DOI:** 10.1371/journal.pone.0029187

**Published:** 2012-01-19

**Authors:** Tatsuo Yamamoto, Tomomi Takano, Wataru Higuchi, Yasuhisa Iwao, Olga Singur, Ivan Reva, Yuta Otsuka, Toru Nakayashiki, Hirotada Mori, Galina Reva, Vladimir Kuznetsov, Vladimir Potapov

**Affiliations:** 1 Division of Bacteriology, Department of Infectious Disease Control and International Medicine, Niigata University Graduate School of Medical and Dental Sciences, Niigata, Japan; 2 Vladivostok State Medical University, Vladivostok, Russia; 3 Graduate School of Biological Sciences, Nara Institute of Science and Technology, Nara, Japan; Swiss Tropical and Public Health Institute, Switzerland

## Abstract

Two distinct classes of methicillin-resistant *Staphylococcus aureus* (MRSA) are spreading in hospitals (as hospital-acquired MRSA, HA-MRSA) and in the community (as community-acquired MRSA, CA-MRSA). Multilocus sequence type (ST) 239 MRSA, one of the most worldwide-disseminated lineages, has been noted as a representative HA-MRSA. Here, we isolated ST239 MRSA (*spa* type 3 [t037] and staphylococcal cassette chromosome *mec* [SCC*mec*] type III.1.1.1) and its novel variant with ST239/*spa*351 (t030)/SCC*mec*III.1.1.4 (SCC*mec*III_R_) not only from hospitals but also from patients with urethritis in the community in Russia. The Russian variant (strain 16K) possessed a hybrid genome consisting of CC8 and CC30, similar to the ST239/*spa*3/SCC*mec*III.1.1.1 HA-MRSA (TW20) genome, but with marked diversity. The 16K′ CC30 section had SCC*mec*III_R_ carrying the *dcs*-carrying unit (which corresponded to the SCC*mec*IVc J3 joining region of ST30 CA-MRSA), lacked SCCmercury, and possessed a novel mobile element structure (MES16K) carrying the *ccrC*-carrying unit (with the recombinase gene *ccrC1* allele 3) and drug resistance tranposons. The Russian variant included strains with a high ability to transfer its multiple drug resistance by conjugation; e.g., for strain 16K, the transfer frequency of a chloramphenicol resistance plasmid (p16K-1 with 2.9 kb in size) reached 1.4×10^−2^, followed by Tn*554* conjugative transfer at 3.6×l0^−4^. The Russian variant, which has been increasing recently, included divergent strains with different plasmid patterns and pulsed field gel electrophoresis profiles. The data demonstrate the alternative nature of ST239 MRSA as CA-MRSA and also as a drug resistance disseminator, and its micro but dynamic evolution in Russia.

## Introduction

Methicillin-resistant *Staphylococcus aureus* (MRSA) was isolated in the early 1960s and has continued to be a life-threatening multiple drug-resistant bacterium in hospitals [1], [2]. MRSA is generated from methicillin-susceptible *S. aureus* (MSSA) by the acquisition of staphylococcal cassette chromosome *mec* (SCC*mec*) at the 3′ end of *orfX* (SCC*mec* insertion site, *att*) [3]. It is considered that this has occurred only a limited number of times, resulting in the current epidemics in hospital settings [3]–[5].

Of these clones, ST239 MRSA is one of the most worldwide-disseminated lineages [4], [6], and is evolutionarily of interest, because it is the first example of a bacterial hybrid, consisting of two distinct MRSA belonging to clonal complex (CC) 30 (founder, ST30) and CC8 (founder, ST8) [7], [8]. The ST239 MRSA lineage exhibits marked geographic variations [6] in terms of the protein A gene (*spa*) type, SCC*mec* type III structures, and pulsed-field gel electrophoresis (PFGE) patterns. Many terms have been used to accommodate this variation [4], e.g., Brazilian clone [9], Portuguese clone [10], [11], Hungarian clone [12]–[14], Viennese clone [15], and British EMRSA-1, -4, and -11 clones [16], [17].

Of those, the Brazilian clone (first ST239 MRSA) spread among hospitals in Brazil in 1992 [18], and then caused intercontinental spread to hospitals in Portugal, possibly linked to Brazil-to-Portugal migration of human populations since 1992–1993 [11], [19]. The Hungarian clone emerged in hospitals in Hungary in 1993, became predominant in hospitals until 1998, and then almost disappeared in 2003–2004 from Hungary [12]–[14]; however, there is no single reference addressing the molecular typing or characteristics of MRSA strains (Hungarian clone) isolated in Hungary. SCC*mec* types were SCC*mec*IIIA for the Brazilian clone (e.g., strain HU25) while SCC*mec*III for the Hungarian clone (e.g., strain HU106) [19].

Moreover, the ST239 TW clone (strain TW20) was noted as intensive care unit (ICU)-associated MRSA in London between 2002 and 2004 [20], and the complete genome of TW20 was described [8]. Harris et al. [6] have recently described the comparative genomics of globally-collected ST239 strains using Illumina genome analysis and the TW20 genome as a reference, demonstrating the global geographic structure within ST239 MRSA, based on genome-wide single nucleotide polymorohisms (SNPs). The ST239 lineage consisted of more than five MRSA clades reflecting the continental origin, such as Asia, North America, South America, Europe (with marked divergence), and Australia, albeit with some intercontinental transmission cases. According to the study of Harris et al. [6], TW20 clustered within the Thai clade (most probably suggesting transmission from southeast Asia to London), the Brazilian clone (e.g., strain HU25) clustered within the South America clade, and Hungarian isolates (during 1993–1996) exhibited divergent European phylogeny in SNPs.

In the community, another class of MRSA, called community-acquired MRSA (CA-MRSA), emerged during the period from 1997 to 1999 [2], [21], [22]. CA-MRSA includes clones belonging to (e.g.) ST8 (USA300), ST30, and ST80 [2], [22], [23]. CA-MRSA generally exhibits SCC*mec*IV or V, a narrow range of drug resistance, and *mecA*-mediated low-level resistance to β-lactam agents (oxacillin and imipenem) [1], [2], [22]–[24].

The term, hospital-acquired MRSA (HA-MRSA), which includes MRSA isolated in the nosocomial environment, has been used since the description of CA-MRSA. In this study, we isolated MRSA, including ST239 MRSA, from patients in hospitals and also from patients with nongonococcal urethritis in the community in Russia, and examined the molecular characteristics of these MRSA strains. We also investigated the comparative genomics of an ST239 variant (strain 16K) with unique features in terms of evolution and drug resistance using the TW20 genome as a reference.

## Materials and Methods

### Patients and bacterial strains

Thirteen outpatients were enrolled in this study, all of whom were male outpatients (age range, 19–40 years; mean age, 29 years) of two hospitals in Vladivostok, Russia, through the period from 2006 to 2008. All outpatients had discharge (pus) from the urethra, discomfort inside the urethra, and discomfort while passing urine. Examination of swabs (discharge) from the urethra revealed the presence of polymorphonuclear neutrophils and MRSA in all cases; no other pathogens, such as *Neisseria gonorrhea*, *Chlamydia*, *Ureaplasma*, *Mycoplasma*, *Candia*, *Trichomonas* and STD-associated viruses, were detected; therefore, outpatients were diagnosed with nongonococcal urethritis due to MRSA. No nosocomial or familial transmissions were observed. The 13 MRSA (including strain 16K) were epidemiologically diagnosed as CA-MRSA; in this study, CA-MRSA was defined as MRSA isolated from outpatients who had no history of hospitalization within at least the past year and presented with no other established risk factors for MRSA infections, such as surgery, residence in a long-term care facility, dialysis, or indwelling percutaneous medical devices and catheters; and HA-MRSA was defined as MRSA isolated from inpatients 48 h after hospitalization [2], [22].

In this study, 18 HA-MRSA strains, isolated from inpatients (age, 34–71 years; mean age, 51.8 years) in four hospitals in Vladivostok between 2004 and 2008, were also examined; they were isolated from surgical wound infections, pneumonia, and blood stream infection (including sepsis). Five HA-MRSA strains were isolated from inpatients (age, 3–64 years; mean age, 26.6 years) in two hospitals in Vladivostok in 2011 from surgical wound infections and burn infections. This study was complied with the ethics review board Niigata University School of Medicine, Niigata, Japan. Written informed consent was obtained from patients.

The epidemic HA-MRSA-type strains included ANS46 (reference strain isolated in 1982 in Australia; ST239/SCC*mec*III), HU25 (Brazilian clone; ST239/SCC*mec*IIIA), BK2464 (New York/Japan clone; ST5/SCC*mec*II), BM18 (Pediatric clone; ST5/SCC*mec*IVa), HDE288 (Pediatric clone; ST5/SCC*mec*VI), HAR22 (EMRSA-15 clone; ST22/SCC*mec*IV), HAR24 (EMRSA-16 clone; ST36/SCC*mec*II), HAR38 (Berlin clone; ST45/SCC*mec*IVa), HPV107 (Iberian clone; ST247/SCC*mec*IA), and COL (Archaic clone; ST250/SCC*mec*I); they were kindly provided by H. de Lencastre. CA-MRSA strain NN1 (ST30/SCC*mec*IVc), which was positive for the collagen adhesin gene (*cna*), was isolated from a child with bullous impetigo in the community [25], [26].

### Genotyping and virulence gene analysis

MRSA typing was performed as described previously [27]. The *spa* type was analyzed by PCR using reference strains, and determined using public *spa* type databases, eGenomics (http://tools.egenomics.com/) or Ridom SpaServer (http://spaserver.ridom.de/). Typing of *agr* was carried out by PCR with previously reported primers [28], [29] or by sequencing the variable region [30]. SCC*mec* types (I to V) were analyzed by PCR using reference strains [31], [32]. The subtypes of SCC*mec*III were described expressing the differences in the J1, J2, and J3 joining regions, according to the guidelines described in 2009 [33]. For virulence gene analysis, the target genes included 46 genes; 3 leukocidin genes (*luk_PV_SF*, *lukE-lukD*, and *lukM*), 5 hemolysin genes (*hla*, *hlb*, *hlg*, *hlg-v*, and *hld*), 18 staphylococcal enterotoxin (SE) genes (*tst*, *sea*, *seb*, *sec*, *sed*, *see*, *seg*, *seh*, *sei*, *sej*, *sek*, *sel*, *sem*, *sen*, *seo*, *sep*, *seq*, and *ser*), 1 putative staphylococcal enterotoxin gene (*seu*), 3 exfoliative toxin genes (*eta*, *etb*, and *etd*), an exotoxin-like gene cluster (*set*), the epidermal cell differentiation inhibitor gene (*edin*), and 14 adhesin genes (*icaA*, *icaD*, *eno*, *fib*, *fnbA*, *fnbB*, *ebpS*, *clfA*, *clfB*, *sdrC*, *sdrD*, *sdrE*, *cna*, and *bbp*) [27].

### PFGE analysis

Bacterial DNA was digested with SmaI and the digested DNA was applied to PFGE (1.2% agarose), as described previously [34]. A lambda ladder (Bio-Rad Laboratories, Tokyo, Japan) was used as the molecular size standard.

### Plasmid analysis

Plasmid DNA of MRSA strains was prepared using a Plasmid Midi Kit (QIAGEN Sciences, Tokyo). Plasmid DNA was introduced into MRSA by electroporation using a Gene Pulser II electroporator (Bio-Rad).

### Conjugative transfer

Donor strains were mated with *S. aureus* RN2677 (recipient strain, which is restriction-negative and resistant to rifampicin and novobiocin) on membrane filters on tryptic soy agar (Difco, Sparks, MD, USA) (filter mating), as previously described [25]; RN2677 was used as a recipient because it carried no plasmids and had non-transmissible drug resistance (recipient) markers. In some experiments, donor and recipient cultures were mixed at 1∶2, centrifuged, and spotted on tryptic soy agar without filters (non-filter mating). Alternatively, donor and recipient cultures were mixed in tryptic soy broth (Difco) (liquid mating). The resistance genes of donor strains and transconjugants were examined by PCR as previously described [27].

### Susceptibility testing

Bacterial susceptibility testing was carried out according to previous procedures [35]. Breakpoints for drug resistance were those described by the CLSI [35].

### Genome analysis

The MRSA 16K genome was analyzed by pyrosequencing using a genome sequencer FLX system with the assembler software GS *De Novo* Assembler version 2.0 (Roche Diagnostics, Branford, CT, USA); pyrosequencing analysis yields contigs of larger sizes than Illumina genome analysis. In this study, 274,241 reads yielded 111 Mb raw sequences, corresponding to approximately 37-fold of the genome size; GenBank accession numbers for the 16K genome (107 contigs with ≥20 bp in size) are BABZ01000001-BABZ01000107. Constructed contigs were mapped on the 3,043,210-bp complete TW20 genome [GenBank accession number FN433596; 6] using MUMmer software (http://mummer.sourceforge.net/). The gene or open reading frame (*orf*) was searched for using the software in silico MolecularCloning (version 4.2) (In Silico Biology, Yokohama, Japan).

### Phylogenetic and homology analysis

Phylogenetic tree analysis was performed using TreeViewX software (version 0.5.0) (http://taxonomy.zoology.gla.ac.uk/rod/treeview.html). The bootstrap value (1000) represents the accuracy of a branch in the phylogenetic tree (analysis was repeated 1,000 times). Homology analysis was performed using the software BLAST (http://blast.ddbj.nig.ac.jp/top-e.html) and FASTA (http://fasta.ddbj.nig.ac.jp/top-j.html).

## Results

### Characteristics of MRSA from outpatients with urethritis in the community

**Figure 1:**
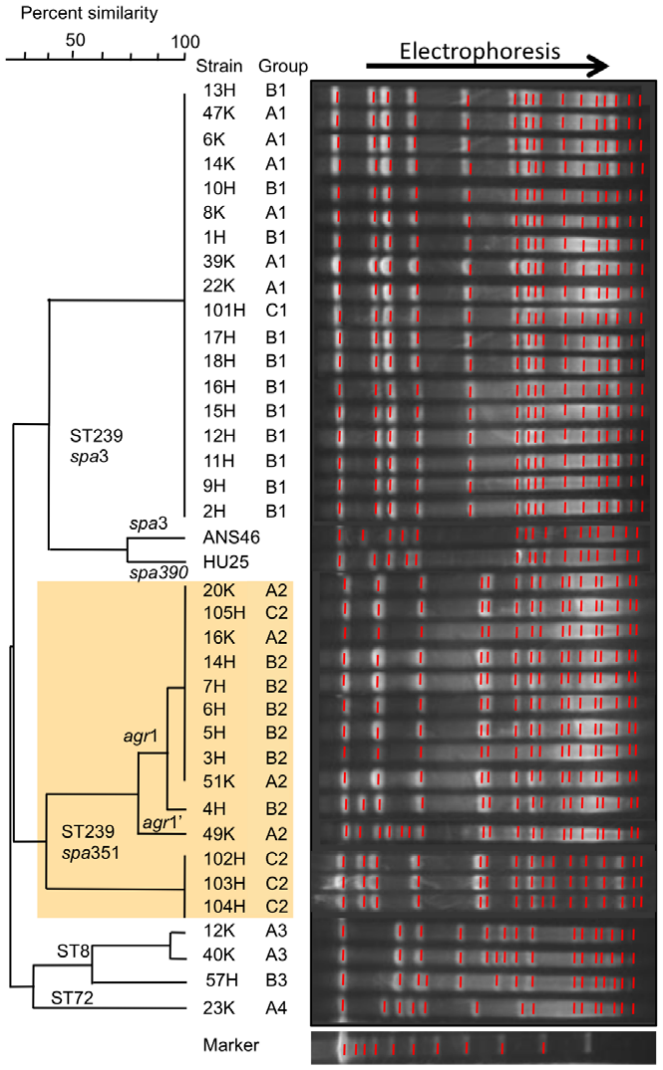
PFGE patterns of MRSA isolated from outpatients with nongonococcal urethritis and from inpatients in Russia compared with ST239 reference strains. MRSA groups (A1–A4, B1–B3, and C1 and C2) are described in Table 1. The Russian variant of ST239 MRSA (groups A2, B2, and C2) is marked with shading (yellow). Strains 6K to 51K (n = 13) are isolated from outpatients during 2006–2008: (group A1) 6K, 8K, 14K, 22K, 39K, 47K; (group A2) 16K, 20K, 49K, 51K; (group A3) 12K and 40K; (group A4) 23K. Strains 1H to 18H (n = 18) are isolates from inpatients during 2004–2008: (group B1) 1H, 2H, 8H, 9H, 11H, 12H, 13H, 15H, 16H, 17H, 18H; (group B2) 3H, 4H, 5H, 6H, 7H, 14H; (group B3) 10H. Strains 101H to 105H (n = 5) are isolates from inpatients in 2011: (group C1) 101H; (group C2) 102H, 103H, 104H, 105H.

Data are summarized in Table 1 and Figure 1. Thirteen MRSA strains were classified into four groups (A1 to A4) (Table 1). Group A1 (n = 6) exhibited ST239/*spa*3/SCC*mec*III.1.1.1 and shared the same genotype with ST239 reference strains, TW20 and ANS46, but was distinct from HU25. Group A1 strains shared the same PFGE pattern (Figure 1).

**Table 1:** Characteristics of MRSA isolated from out patients with nongonococcal urethritis and from inpatients in Russia compared with ST239 MRSA reference strains.

	MRSA from urethritis(CA-MRSA)2006–2008				MRSA from inpatients (HA-MRSA)2004–2008			MRSA from inpatients (HA-MRSA) 2011		ST239 MRSA reference strains		
Type, virulence gene or drug resistance	Group A1	Group A2	Group A3	Group A4	Group B1	Group B2	Group B3	Group C1	Group C2	TW20^a^ UK 2003	ANS46 Australia 1982	HU25 Brazil 1993
Type	(n = 6)	(n = 4)	(n = 2)	(n = 1)	(n = 11)	(n = 6)	(n = 1)	(n = 1)	(n = 4)			
CC	8	8	8	8	8	8	8	8	8	8	8	8
ST	239	239	8	72	239	239	8	239	239	239	239	239
*spa*	3 (t037)	351 (t030)	826 (tUK)	451 (t324)	3 (t037)	351 (t030)	826 (tUK)	3 (t037)	351 (t030)	3 (t037)	3 (t037)	390 (t138)
*agr*	1	1 (3/4), 1′ (1/4)	1	1	1	1	1	1	1	1	1	1
SCC*mec* type	III.1.1.1	III.1.1.4 (III_R_)	IVc	IVc	III.1.1.1	III.1.1.4 (III_R_)	IVc	III.1.1.1	III.1.1.4 (III_R_)	III.1.1.1	III.1.1.1	III.1.1.2 (IIIA)
Coagulase type	IV	IV	III	V	IV	IV	III	IV	IV	IV	IV	IV
Virulence gene												
Leukocidin												
*lukE-lukD*	+	+	+	+	+	+	+	+	+	+	+	+
Hemolysin												
*hla, hlb (split), hlg, hlg-v*	+	+	+	+	+	+	+	+	+	+	+	+
*hld*	+	+	+	+	+	+	+	+	+ (1/4)	+	+	+
Enterotoxin												
*egc* (*seg, sei, sem, sen, seo*)	−	−	−	+	−	−	−	−	−	−	−	−
*sea*	+	+	+	−	+	+	+	+	+	+	+	−
SaPI1 (*sek, seq*)	+	+	−	−	+	+	−	+	+	+	+	−
Adhesin												
*c12ag* b	+	+	+ (1/2)	+	+	+	+	+	+	+	+	+
*cna*	+	+	−	−	+	+	−	+	+	+	+	+
Susceptibility^c^												
Oxa (MIC, µg/ml)	≥256	≥256	32	32	≥256	≥256	64	≥256	≥256	ND	128	≥256
Ipm (MIC, µg/ml)	32–64	64	0.06	0.5	32–64	64–128	1	32	64–128	ND	16	64
Resistance to non β-lactam	Gen, Kan, Str, Spt, Tet, Ery, Cli, Lvx, Sul, Tmp	Gen, Kan, Str, Spt, Tet, Ery, Cli, Lvx, Sul, Chl, Rif	Gen, Kan, Ery (1/2), Cli (1/2), Chl	Kan	Gen, Kan, Str, Spt, Tet, Ery, Cli, Lvx, Sul (10/11), Tmp, Chl (1/11)	Gen, Kan, Str, Spt, Tet, Ery, Cli, Lvx(R, 4/6; I 2/6), Sul, Chl, Rif	Gen, Kan, Ery, Cli, Chl, Rif	Gen, Kan, Str, Spt, Tet, Ery, Cli, Lvx, Sul, Tmp	Gen, Kan, Str, Spt, Tet, Ery, Cli (1/4), Lvx, Sul, Chl, Rif	Gen, Kan, Str, Tet, Ery, Cli, Lvx, Tmp	Kan, Str, Spt, Tet, Ery, Cli, Sul, Tmp, Chl	Gen, Kan, Str, Tet, Ery, Cli, Lvx, Sul, Tmp
Plasmids^d^ (kb)	38* (5/6), 2.9 (4/6)	32 (1/4), 4.4 (1/4), 2.9** (d), 2.4	34, 2.9**, 2.6 (1/2)	3.6, 2.7	41** (1/11),38 (7/11), 2.9 (10/11), 2.6 (1/11)	41** (5/6), 30** (1/6), 2.9 (d), 2.4	-	38, 2.9	41** (1/4) 38*(3/4), 2.9 (3/4)** (d), 2.4	29.6*, 3.0	4.4*	-

MRSA groups A2, B2, and C2 represent the Russian variant of ST239 MRSA.

a The data of TW20 are from GenBank accession numbers FN433596, FN433597, and FN433598. A 29.6-kb plasmid (pTW20_1) is a heavy metal resistance plasmid [8].

b *c12ag*, core 12 adhesin genes shared by all (or most) strains: *icaA*, *icaD* (for biofilm formation); *eno* (for laminin-adhesin); *fnbA*, *fnbB* (for fibronectin-adhesin); *ebpS* (for elastin-adhesin); *clfA*, *clfB*, *fib*, *sdrC*, *sdrD*, *sdrE* (for fibrinogen).

c Oxa, oxacillin; Imp, imipenem; Gen, gentamicin; Kan, kanamycin; Str, streptomycin; Spt, spectinomycin; Ery, erythromycin; Cli, clindamycin; Tet, tetracycline; Lvx, levofloxacin; Sul, sulfamethoxazole; Tmp, trimethoprim; Chl, chloramphenicol; Rif, rifampicin. MICs of rifampicin: 256 µg/ml for groups A2, B2, and C2; 8 µg/ml for group B3. ND, not determined.

d Resistance plasmids were examined by conjugation and electroporation.

*, plasmid encoding for cadmium resistance;

**, plasmid encoding for chloramphenicol resistance.

In group A2, strain 49K carried 32-kb and 4.4-kb plasmids in addition to 2.9-kb and 2.4-kb plasmids. (−), no plasmid.

Group A2 (n = 4) was a variant of the ST239 lineage. These strains (including 16K) exhibited *spa*351/SCC*mec*III.1.1.4 (named SCC*mec*III_R_), were resistant to chloramphenicol and rifampicin and susceptible to trimethoprim, and possessed a 2.9-kb chloramphenicol-resistance plasmid (Table 1). Group A2 strains shared the same PFGE pattern, except for one strain (49K) (Figure 1); 49K exhibited a divergent *agr* subtype (named *agr1'*; see Fig. S1 for detailed analysis) and plasmid profile (Table 1).

Group A3 (n = 2; ST8/SCC*mec*IVc) and group A4 (n = 1; ST72/SCC*mec*IVc) exhibited a narrow range of drug resistance and low oxacillin and imipenem resistance levels (Table 1), similarly to CA-MRSA [24].

The ST239 lineage strains, but not ST8 or ST72 strains, were positive for the collagen adhesin gene (*cna*). Of the epidemic-type HA-MRSA clones, EMRSA-15 (ST22), EMRSA-16 (ST36), and Berlin (ST45) were also *cna*-positive.

### Characteristics of MRSA from inpatients

Data are summarized in Table 1 and Figure 1. Of 18 MRSA strains epidemiologically diagnosed as HA-MRSA during 2006–2008, 11 strains belonged to group B1 (ST239/*spa*3/SCC*mec*III.1.1.1) and corresponded to group A1; one strain (strain 2H) possessed a 41-kb chloramphenicol-resistance plasmid. Six strains belonged to group B2 (ST239/*spa*351/SCC*mec*III_R_) and corresponded to group A2, although most B2 strains possessed a 41-kb chloramphenicol-resistance plasmid. The remaining strain belonged to group B3 (ST8/SCC*mec*IVc) and resembled group A3.

Therefore, around 2006, the ST239/*spa*3/SCC*mec*III.1.1.1 type was the most prevalent and the variant (ST239/*spa*351/SCC*mec*III_R_) type was the second most prevalent, not only in the community but also in hospitals.

Of the five MRSA strains epidemiologically diagnosed as HA-MRSA in 2011, one strain (group C1) corresponded to group A1 or B1. Four strains (group C2) exhibited the same genotype as group A2 or B2, indicating the increase of the ST239 variant type in 2011; however, of four C2 strains, three strains exhibited slightly distinct PFGE profiles and plasmid patterns (containing 2.9-kb chloramphenicol resistance and 38-kb cadmium resistance plasmids) (Table 1 and Figure 1).

### Comparative genomics of the ST239 Russian variant (strain 16K)

**Figure 2:**
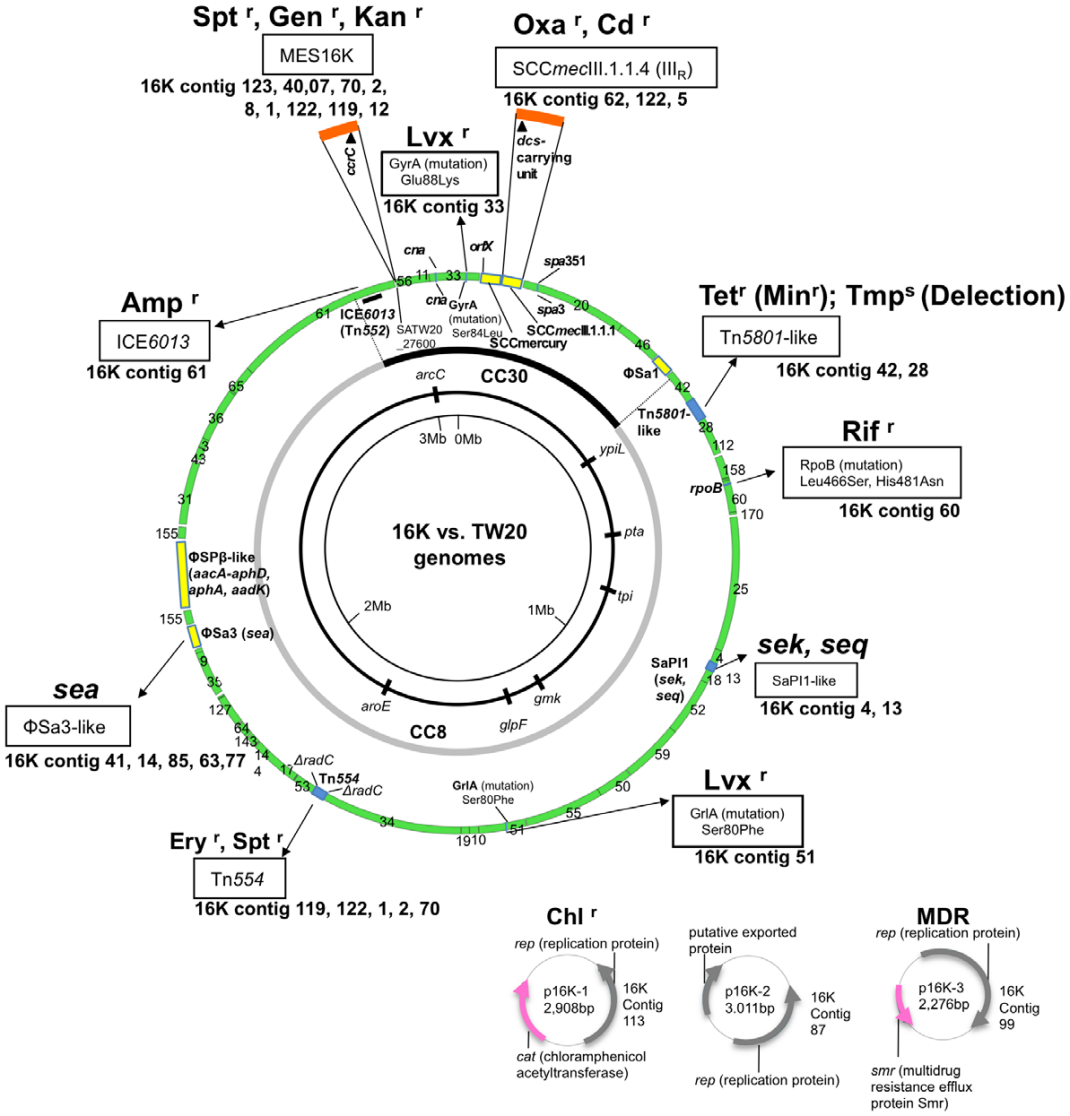
Genome information for ST239 MRSA strain 16K in comparison with ST239 MRSA strain TW20. The genome information includes drug resistance genetic traits (related structures and mutations), virulence genes, superantigen-associated phages, and plasmids. The 16K genome contigs, obtained by pyrosequencing, were mapped on the 3,043,210-bp TW20 genome (GenBank accession number FN433596; shown as a circle). The information on the 16K and TW20 genomes is presented outside and inside the genome circle, respectively. Colored regions in the 16K genome map: green, highly homologous to TW20; yellow, non- or less homologous to TW20; blue, slightly divergent from TW20; brown, insertion. Drug resistance (r): Oxa, oxacillin; Tet, tetracycline; Min, minocycline; Rif, rifampicin; Lvx, levofloxacin; Chl, chloramphenicol; Ery, erythromycin; Spt, spectinomycin; Gen, gentamicin; Kan, kanamycin; Amp, ampicillin; Ery, erythromycin. MDR, multiple drug resistance. The three plasmids of strain 16K are shown at the right bottom of the figure. The CC30 and CC8 genome sections are from Holden *et al.* [8].

The 16K genome sequence was compared with the TW20 genome (Figure 2). The 16K genome was estimated to be at least 2.8 Mb in size, showing approximately 99.9% homology with the TW20 region (in green), excluding four large non- or less homologous regions (283.6-Kb, in yellow). The CC30 and CC8 hybrid regions on the genome were assigned according to Holden *et al*. [8].

In the CC30 section, 16K possessed SCC*mec*III_R_ instead of SCC*mec*III.1.1.1. Moreover, 16K lacked SCCmercury, but possessed a novel mobile element structure (MES16K), which carried the recombinase gene (*ccrC*)-carrying unit [36], similarly to SCCmercury.

There were several phage deletions in16K: ΦSa1 and ΦSPβ-like were absent; and a part of ΦSa3 was lacking (Figure S2A). Moreover, marked divergence was observed with SaPI1 (Figure S2B).

In 16K, the *blaz* gene (encoding ampicillin resistance) was located in ICE6013 (Figure 2), and the *tetM* gene (encoding tetracycline and minocycline resistance) was in a smaller Tn*5801*-like structure, which lacked *dfrG* (encoding trimethoprim resistance) (Figure S2C). Rifampicin resistance (MIC, 256 µg/ml) was due to Leu 466Ser and His481Asn mutations in *rpoB*, and levofloxacin resistance (MIC, 4 µg/ml) was due to mutations Ser80Phe in *grlA* and Glu88Lys in *gyrA* (Figure 2).

Strain 16K possessed three plasmids, 2.9-kb chloramphenicol resistance plasmid (p16K-1), 3.0-Kb putative exported protein plasmid (p16K-2), which corresponded to 3.0-Kb pTW20_2 of TW20, and 2.3-Kb multidrug resistance efflux protein plasmid (p16K-3), which showed similarity to 2.4-Kb pPSK108 of *S. epidermidis*.

### SCC*mec*III_R_ structure of strain 16K

**Figure 3:**
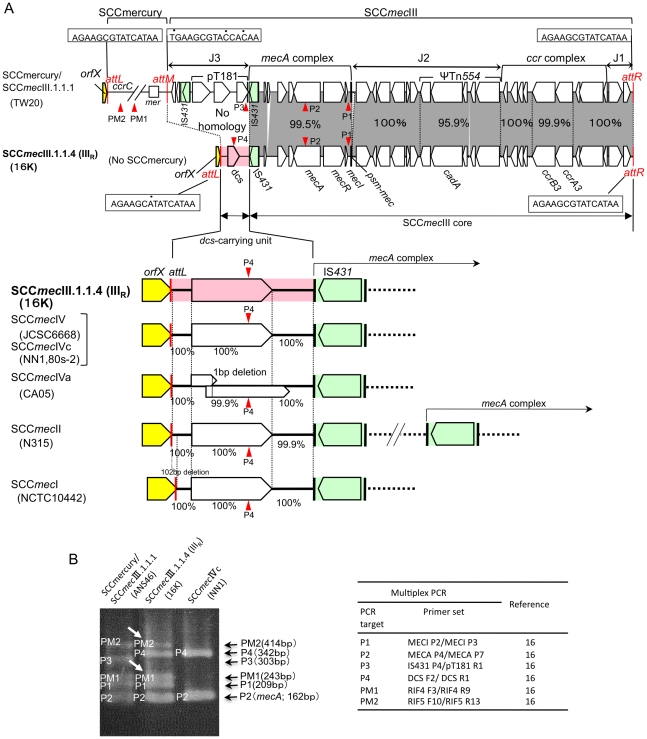
The SCC*mec*III_R_ structure of ST239 MRSA strain 16K. GenBank accession number for SCC*mec*III_R_ is AB539727. The data for strain TW20 are from GenBank accession number FN433596. Homologous regions are shaded. In A, when the structure of SCC*mec*III_R_ was compared with SCCmercury/SCC*mec*III.1.1.1 of TW20 (upper part of figure), the SCC*mec*III core was shared by the two, but the left side regions (flanked by *att* and IS*431*) of SCC*mec*III_R_ and SCC*mec*III.1.1.1 showed no homology. Moreover, Strain 16K lacked SCCmercury. The *dcs*-carrying unit (marked in pink) was shared by SCC*mec*III_R_, SCC*mec*IV, SCC*mec*II, and SCC*mec*I (lower part of figure). The data for strains JCSC6668, NN1, 80s-2, CA05, N315, and NCTC10442 were from GenBank accession numbers AB425823, AB245470, AB245471, AB063172, NC_002745, and AB033763, respectively. In B, the mosaic SCC*mec*III_R_ structure was specifically detected by multiplex PCR. Multiplex PCR detects targets: P1 and P2 in the SCC*mec*III core, P3 in the left side region (attM-pT181) of SCC*mec*III.1.1.1, P4 in the left side region (*dcs*-carrying unit) of SCC*mec*III_R_, and PM1 (in an *orf*) and PM2 in SCCmercury or MES16K (PCR targets are shown in this figure [in A] and Fig. 4 for SCCmercury, and in Fig. 4 for MES16K); strain 16K was negative for the *mer* operon (lacked SCCmercury). ST239/SCC*mec*III.1.1.1 MRSA strain ANS46 gave five PCR bands; three bands (P1, P2, and P3) for SCC*mec*III.1.1.1 and two bands (PM1 and PM2) for SCCmercury. In contrast, the Russian variant (strain 16K) gave different combinations of five PCR bands; three bands (P1, P2, and P4) for SCC*mec*III_R_ and two bands (PM1 and PM2, marked with arrows) for MES16K. PCR band P4 was shared by SCC*mec*III_R_ and SCC*mec*IVc (and SCC*mec*IVa, SCC*mec*II, and SCC*mec*I). The combination of P1, P2, P4, PM1, and PM2 was unique to the Russian variant (strain 16K).

The complete sequence of 16K SCC*mec*III_R_ was determined (Figure 3A). It was 31,198 bp in size, and consisted of 15-bp *attL* (adjacent to *orfX*), 2,208-bp *dcs*-carrying unit (J3 region), 28,960-bp SCC*mec*III core region (carrying *mecA* complex and *ccr* complex), and 15-bp *attR*. The J3 region of 16K showed no homology with the 6,266-bp J3 region of TW20. The *dcs*-carrying unit (J3) was shared by 16K SCC*mec*III_R_, SCC*mec*I, SCC*mec*II, and SCC*mec*IV (Figure 3A); of those, the sequences of 16K SCC*mec*III_R_ and SCC*mec*IVc (of ST30 CA-MRSA strains NN1 and 80s-2) were identical, suggesting that the *dcs*-carrying unit (J3) of 16K SCC*mec*III_R_ was acquired by recombination. The mosaic SCC*mec*III_R_ structure was identified by multiplex PCR (Figure 3B), although PCR (PM1 and PM2) detected MES16K in strain 16K (which lacked SCCmercury).

### MES16K structure of strain 16K

**Figure 4:**
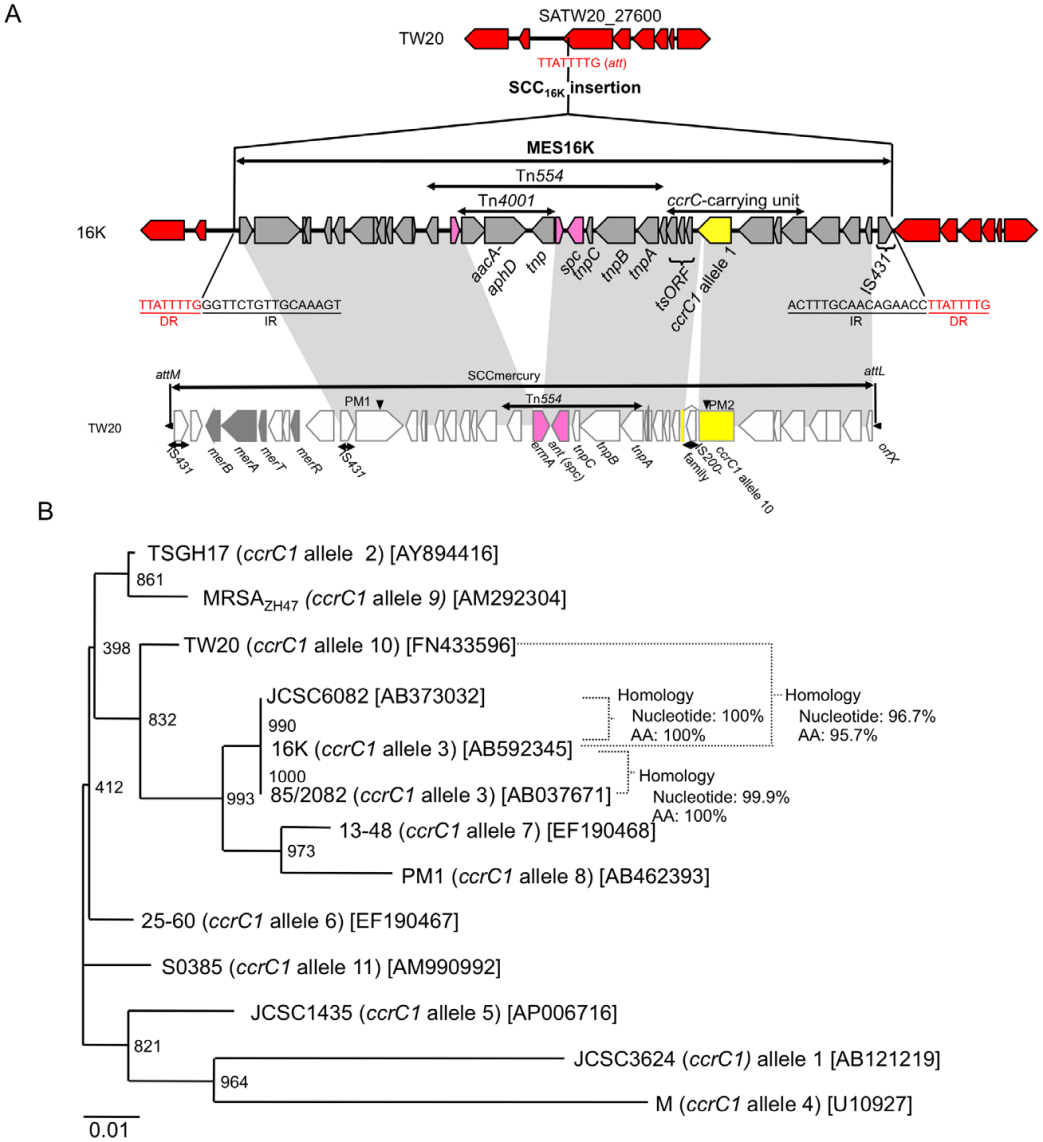
MES16K structure and phylogenetic tree analysis for the *ccrC* genes. The SCCmercury data for strain TW20 are from GenBank accession number FN433596. The GenBank accession number of MES16K is AB666466. Homologous regions are shaded. In A, for strain TW20, the *ccrC*-carrying unit was located within SCCmercury; the *ccrC* gene (marked in yellow) was split by the insertion of IS (IS200 family). For strain 16K, the *ccrC*-carrying unit was located within a mobile element structure MES16K, which was inserted into the *orf* (corresponding to SATW20_27600 of TW20) on the 16K genome, as shown in Fig. 2; the *ccrC* gene (marked in yellow) was intact. The attachment sequence (*att*) of MES16K showed no homology to that (*att*) of the *orfX*, SCC*mec*III.1.1.1 or SCCmercury. Arrowheads PM1 and PM2 represent PCR targets for detection of SCCmercury of ST239/SCC*mec*III.1.1.1 MRSA and MES16K of the Russian variant (strain 16K) by multiplex PCR (Fig. 3). In B, the *ccrC* gene sequence of strain 16K and that of strain TW20 (expected intact *ccrC* gene sequence, obtained by excluding the inserted IS200-family sequence) were analyzed for phylogenetic diversity, as described previously [36]. The *ccrC* gene of strain TW20 was a novel *ccrC1* allele (named *ccrC1* allele 10) and that of strain 16K was assigned as *ccrC1* allele 3, unambiguously demonstrating evolutionary diversity between them; moreover, the *ccrC1* gene of ST398 MRSA strain S0385 was also a novel allele (named *ccrC1* allele 11).

The complete sequence of MES16K was determined (Figure 4A). It was 30,818 bp in size and flanked by 16-bp inverted repeats (IRs). MES16K was inserted into the *orf* on the 16K genome, corresponding to SATW20_27600 of TW20; the *att* (target) sequence was 8-bp long, and was duplicated at both ends of MES16K. The 8-bp *att* and 16-bp IR sequences showed no homology to the 15-bp *att* sequences for SCC*mec*III or SCCmercury. MES16K shared a similar *ccrC*-carrying unit with SCCmercury, albeit with *ccrC1* allele 3 for 16K and *ccrC1* novel allele 10 for TW20 (Figure 4B) and also with IS insertion in *ccrC1* in TW20 (but not in 16K). Tn*554* was present in both MES16K and SCCmercury, but in MES16K, Tn*4001* was inserted into the *ermA* gene of Tn*554*.

### Conjugative transfer of drug resistance

The ST239/*spa*351/SCC*mec*III_R_ variant (especially group A2) transferred its drug resistance to *S. aureus* RN2677 by bacterial mating on agar plates, in the order of chloramphenicol, erythromycin, gentamicin, cadmium, and tetracycline resistance (Table 2).

**Table 2:** Conjugative transfer of drug resistance from Russian MRSA strains to *S. aureus* RN2677.

Donor MRSA strains^a^	Strains examined (n)	Transfer frequency^b^: drug-resistant recipients/donor					
		(recipient, RN2677)					
		Chloramphenicol resistance	Erythromycin resistance	Gentamicin resistance	Tetracycline resistance	Oxacillin resistance	Cadmium resistance
Russian isolates							
Group A1	6	–c	<1.0×10^−9^	<1.0×10^−9^	<1.0×10^−9^	<1.0×10^−9^	<1.0×10^−9^
(ST239/*spa*3)							
Group A2	4	**1.4×10^−2^ –**	**3.6×10^−4^ –**	**1.0×10^−5^ –**	**2.8×10^−8^ –**	<1.0×10^−9^	**2.1×10^−6^ –**
(ST239/*spa*351)		**5.3×10^−4^** d	**6.7×10^−5^**	**6.9×10^−6^**	**5.6×10^−9^**		**1.8×10^−7^**
		**(** ***cat*** **/p2.9)**	**(** ***ermA*** **)**	**(** ***aacA-aphD*** **)**	**(** ***tetM*** **)**		**(** ***cadA*** **)**
Group A3	2	**9.5×10^−5^ –**	**1.8×10^−5^**	<1.0×10^−9^	–c	<1.0×10^−9^	<1.0×10^−9^
(ST8/*spa*826)		**3.0×10^−6^**	**(** ***ermA*** **)**				
		**(** ***cat*** **/p2.9)**					
Group A4	1	–c	–c	–c	–c	<1.0×10^−9^	<1.0×10^−9^
(ST72/*spa*3451)							
Group B1							
(ST239/*spa*3)	10	–c	<1.0×10^−9^	<1.0×10^−9^	<1.0×10^−9^	<1.0×10^−9^	<1.0×10^−9^
(ST239/*spa*3)	1^e^	**2.3×10^−3^**	<1.0×10^−9^	<1.0×10^−9^	<1.0×10^−9^	<1.0×10^−9^	<1.0×10^−9^
		**(−/p41)**					
Group B2	6	**1.1×10^−2^ –**	**4.2×10^−7^ –**	**3.7×10^−8^ –**	<1.0×10^−9^	<1.0×10^−9^	<1.0×10^−9^
(ST239/*spa*351)		**9.7×10^−3^**	**1.6×10^−8^**	**1.6×10^−8^**			
		**(−/p41 or p30)**	**(** ***ermA*** **)**	**(** ***aacA-aphD*** **)**			
Group C1	1	–c	<1.0×10^−9^	<1.0×10^−9^	<1.0×10^−9^	<1.0×10^−9^	<1.0×10^−9^
(ST239/*spa*3)							
Group C2							
(ST239/*spa*351)	1^f^	**6.5×10^−3^**	**5.5×10^−4^**	**5.7×10^−5^**	<1.0×10^−9^	<1.0×10^−9^	3.9×10^−7^
		**(−/p41)**	**(** ***ermA*** **)**				
(ST239/*spa*351)	3^g^	**3.3×10^−6^ –**	**5.2×10^−7^ –**	<1.0×10^−9^	<1.0×10^−9^	<1.0×10^−9^	**6.4×10^−7^ –**
		**1.2×10^−7^**	**2.0×10^−7^**				**1.5×10^−7^**
		**(** ***cat*** **/p2.9)**	**(** ***ermA*** **)**				**(** ***cadA,cadD/p38*** **)**
ST239 reference strains							
ANS46		<1.0×10^−9^	**9.5×10^−8^**	–c	<1.0×10^−9^	<1.0×10^−9^	<1.0×10^−9^
			**(** ***ermA*** **)**				
HU25		–c	**6.0×10^−6^**	<1.0×10^−9^	<1.0×10^−9^	<1.0×10^−9^	<1.0×10^−9^
			**(** ***ermA*** **)**				

a Groups of MRSA strains from Russia are from Table 1.

b Drug resistance genes acquired by transconjugants are shown in parentheses (*cat*, chloramphenicol resistance; *ermA*, erythromycin and clindamycin resistance; *aacA-aphD*, gentamicin and kanamycin resistance; *tetM*, tetracycline and minocycline resistance; *cadA*, cadmium resistance; −, no previously-described sequences). Plasmid size was described after the resistance gene in parentheses.

c Donor MRSA strains were susceptible to each drug (resulting in no transconjugants).

d Resistance transfer frequencies (average data in two or three experiments) for four group2 isolates were 1.4×10^−2^, 6.5×10^−3^, 1.0×10^−3^, and 5.3×10^−4^.

e, f, g Strain 2H; 105H; and 102H, 103H, and 104H, respectively.

Plasmids were detected only in chloramphenicol-resistant transconjugants (Tables 1 and 2). For group A2 MRSA (isolated from the community during 2006–2008), the size of chloramphenicol-resistance plasmids was 2.9-kb. The sequence of the chloramphenicol-resistance plasmid (GenBank accession number, AB539725), donated from strain 16K (group A2), was consistent with p16K-1 in Figure 2. In contrast, for MRSA strains from hospitals during 2004–2008 (one B1 strain [strain 2H] and B2 strains), the plasmid size was 41-kb or 30-kb. And, for MRSA from hospitals in 2011, the plasmid size in one strain (strain 105H) was 41-kb, while the plasmid size in three strains (strains 102H, 103H, and 104H) was 2.9-kb, indicating periodical changes of chloramphenicol-resistance plasmids.

No transfer or low-level transfer was observed for ST239/*spa*3/SCC*mec*III.1.1.1 strains (groups A1, B1, and C1), except for strain 2H (group B1) which donated a 41-kb chlorampenicol resistance plasmid (Table 2). Moreover, group A1 strain 6K carrying p16K-1, which was constructed by introduction of p16K-1 DNA into strain 6K by electroporation, gave no transconjugants, indicating that p16K-1 transfer occurred in a host (strain 16K)-dependent manner.

**Figure 5:**
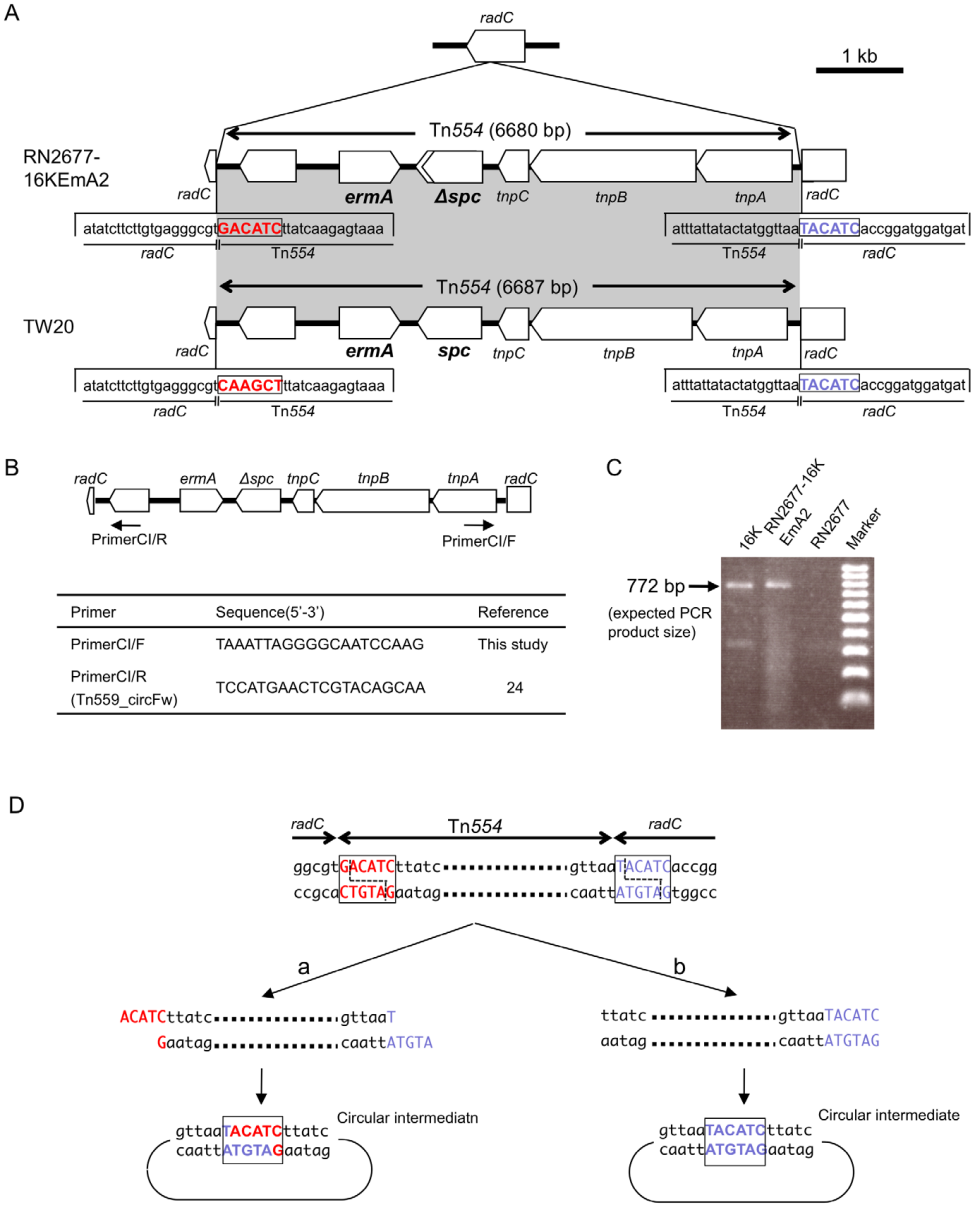
The structure of Tn*554* and its circular intermediate in erythromycin-resistant transconjugants (RN2677-16KEmA2). Transconjugant RN2677-16KEmA2 was obtained by mating between strain 16K (donor) and *S. aureus* RN2677 (recipient). The data for strain TW20 are from GenBank accession number FN433596. In A, Tn*554* was integrated into the *radC* gene in RN2677, at exactly the same site as the Tn*554* integration site in *radC* for strain TW20; the *radC* right-side hexanucleotide sequence, immediately adjacent to Tn*554*, is marked in blue. The Tn*554* left-side hexanucleotide sequence (marked in red) was distinct between the two Tn554 sequences of strains RN2677-16KEmA2 and TW20, reflecting previous target site sequences for each Tn*554* (note that the orientation of the Tn*554* structure in the figure is opposite, when compared with previously described Tn*554* structures [37]–[39]). The *spc* gene on Tn*554* in a transconjugant (RN2677-16KEmA2) had a single base substitution, resulting in a truncated gene product (lacking 27-aa at the 3′-end); the mutated *spc* gene still conferred spectinomycin resistance. In B, since Tn*554* transposes through a circular intermediate [38]–[39], PCR primers to detect a Tn*554* circular intermediate were designed for Tn*554* originated in strain 16K. In C, the PCR primer set (CI/F and CI/R, shown in B) exactly detected a Tn*554* circular intermediate in strains 16K and RN2677-16KEmA2 (PCR product size, 772 bp) in PCR assay; there were no such bands for strain RN2677. In D, when the sequence of the PCR product, obtained in C, was determined, the Tn*554* circular intermediate in RN2677-16KEmA2 possessed the *radC* hexanucleotide (5′-TACATC; marked in blue), but not the Tn*554* hexanucleotide sequence (5′-GACATC; marked in red).

In mating between 16K and RN2677, erythromycin-resistant transconjugants (including RN2677-16KEmA2) possessed Tn*554* (GenBank accession number, AB539726) inserted into the *radC* gene (Figures 2 and 5A). There were no repeats at either terminus of Tn*554* and no repeats of the “target” sequence, unlike other transposons (as reported previously [37]–[39]). The hexanucleotide sequence at the left end of Tn*554* in RN2677-16KEmA2 (5′-GACATC) was distinct from that of Tn*554* within the TW20 *radC* gene (5′-CAAGCT) (Figure 5A; sequence in red), reflecting the former target site sequence [37]–[39] in each case. The free circular intermediate of Tn*554* was present for both donor strain 16K and transconjugant RN2677-16KEmA2 (Figure 5B–D). For Tn*554* in the transconjugant, there was a deletion in *spc* (Figure 5A), although it still conferred spectinomycin resistance.

Conjugative transfer of erythromycin resistance was also observed for group A3, group B2, group C2, and ST239 reference strains ANS46 and HU25 (Table 2); these transconjugants also carried spectinomycin resistance gene (*spc*) and were positive for a Tn*554* circular intermediate by PCR (data not shown).

## Discussion

The ST239 MRSA lineage is a representative multiple drug-resistant HA-MRSA circulating worldwide [2], [4]–[6], [8]; for example, it includes TW20, which emerged in an ICU in London [20]. Harris et al. [6] recently demonstrated, based on genome-wide SNPs, that the ST239 lineage consisted of more than five MRSA clades, such as Asia, North America, South America, Europe, and Australia. In this study, we isolated two divergent ST239 MRSA groups with *spa*3/SCC*mec*III.1.1.1 and *spa*351/SCC*mec*III_R_ from Russia; the latter has tended to increase recently. According to Harris et al. [6], *spa*3 (t037) represents the ancestral (most prevalent) ST239 *spa* type, and the *spa*3 type is distributed worldwide, including North and South America, Australia, Europe, and Asia. In contrast, the *spa*351 (t030) type has been found only in areas of Europe. Based on this, together with the deletion patterns of genomic islands and mobile genetic elements by Harris et al. [6], the Russian ST239/*spa*351/SCC*mec*III_R_ variant may belong to the European clade, suggesting possible transmission from Europe to Far Eastern Russia (although SCC*mec*III subtypes or sequences have not been described by Harris et al. [6]).

The two ST239 MRSA groups in Russia were isolated not only from patients in hospitals but also from patients with nongonococcal urethritis in the community; therefore, MRSA may be an agent of sexually transmitted disease in Russia, which would cause great concern about the potential to spread in the community. When the Russian ST239 MRSA strains (*spa*3/SCC*mec*III.1.1.1 or *spa*351/SCC*mec*III_R_) from hospitals and the community were compared, there was a marked divergence in plasmid patterns; e.g., a 2.9-kb chloramphenicol-resistance plasmid in group A2 vs. a 41-kb chloramphenicol-resistance plasmid in group B2. Moreover, the ST239/*spa*351/SCC*mec*III_R_ variant isolated recently (in 2011) showed a slightly divergent PFGE pattern, suggesting micro but dynamic evolution in Russia.

The CC30 genome section of the ST239 hybrid genome (16K) carried two mobile elements, SCC*mec*III_R_ and MES16K, and three potential virulence genes, *spa* [40], [41], *cna* [26], [42], and *psm-mec* (encoding a peptide cytolysin [43]). SCC*mec*III_R_ was a mosaic SCC*mec*, consisting of the SCC*mec*III core and *dcs*-carrying unit (as a J3 region). An identical *dcs*-carrying unit was found in SCC*mec*IVc of ST30 CA-MRSA (strains NN1 and 80s-2 in Japan [25], [44] and also strain RS08 in Russia [45]), indicating that the *dcs*-carrying unit of the Russian variant could originate in ST30 CA-MRSA. The *dcs*-carrying unit corresponds to a region with the *ccrC*-carrying unit in SCC*mec*VII [36] (now renamed SCC*mec*V [33]). This region flanked by *orfX*(*attL*) and IS*431* seems to be a hot spot for recombination.

In strain 16K, the *ccrC*-carrying unit, found in SCC*mec*V and SCCmercury [36], was located in MES16K, which carried a composite transposon. MES16K is markedly distinguished from SCC*mer* or SCCmercury, even in terms of the attachment sequence (*att*). The *ccrC* gene is highly variable; for example, it is *ccrC1* allele 3 for strain 16K, *ccrC1* allele 8 for ST59 CA-MRSA strain PM1 [36], and *ccrC1* allele 10 (novel allele type) for ST239 HA-MRSA strain TW20.

One of the most striking features of the Russian variant is its behavior as a multiple drug resistance disseminator. To our knowledge, this is the first demonstration of multiple drug resistance transfer in the ST239 MRSA lineage. For mating, close contact of donor and recipient cells on agar plates is needed, but the use of filters is not essential. There is a possibility that during close-contact mating, plasmids in donor (16K) cells are preferably transferred into recipient bacterial cells, as in the case of p16K-1. For Tn*554*, which transposes through the circular intermediate [38], [39], the circular intermediate may be transferred, followed by subsequent integration into the recipient genome, thus exhibiting lower transfer frequency than a chloramphenicol plasmid (p16K-1).

In conclusion, the ST239/*spa*3/SCC*mec*III.1.1.1 MRSA and its variant (ST239/*spa*351/SCC*mec*III_R_) have emerged not only in hospitals but also in the community, associated with nongonococcal urethritis, in Russia. The Russian variant showed high similarity to TW20 in terms of the bulk of the hybrid genome, but was markedly divergent from TW20, such as *spa*351 vs. *spa*3, novel mosaic SCC*mec*III_R_ vs. SCC*mec*III.1.1.1, MES16K vs. SCCmercury, and the presence vs. absence of small chloramphenicol-resistance plasmids. The Russian variant behaved as a drug resistance disseminator, mediating chloramphenicol-resistance plasmid transfer and conjugative Tn*554* transposition. Micro but dynamic evolution of ST239 MRSA is proceeding in Russia.

## Supporting Information

Figure S1 The *agr* subtyping for the Russian variant strain 49K. In A, PCR primers for *agr* typing are shown as an arrow. Strains: agr1, *agr*1 type strain; agr2, *agr*2 type strain; 49K, the Russian variant strain 49K. Strain 49K produced a product only for primer sets (Pan and agr1), and not for (Pan and agr2), assigning strain 49K as *agr*1. However, in the other PCR assay, strain 49K produced a product both for primer sets (agr1 forward and agr1 reverse) and for agr2 forward and agr2 reverse, resulting in no precise assignment. In B, the *agr* subtypes were examined by phylogenetic tree analysis, based on the nucleotide sequences of the *agr* variable region, as described previously [30]. GenBank accession number for the variable region sequence of strain 49K is AB592345. Based on the data, strain 49K was assigned as agr1′.
(TIF)

Figure S2 Deletions in drug resistance or virulence genetic traits in 16K, in comparison with TW20. The data of strain TW20 are from GenBank accession number FN433596. Homologous regions are shaded. In A, contigs 41, 14, 85, and 63 (and probably 77) of strain 16K constituted the bulk of the ΦSa3 phage sequence, showing the presence of the *sea* gene (encoding superantigen SEA, a virulence factor associated with severe invasive infections) in strain 16K; no right side sequence of ΦSa3 was present. In B, contigs 4 and 13 of strain 16K constituted the left and right side regions of the SaPI1 superantigen-associated pathogenicity island, showing the presence of the superantigen genes *sek* and *seq* in strain 16K. In C, contigs of 42 and 28 of strain 16K constituted the Tn*5801*-like sequence (including 20-bp direct repeat at both ends), but with deletion of the *dfrG* gene sequence (encoding trimethoprim resistance) in contig 28. Strain 16 was also negative for *dfrG* by PCR assay.
(TIF)
