# Correction: Comparative Genomics and Drug Resistance of a Geographic Variant of ST239 Methicillin-Resistant *Staphylococcus aureus* Emerged in Russia

**DOI:** 10.1371/annotation/05a851e1-8627-4ea4-b12a-cfdba09f1e61

**Published:** 2012-08-06

**Authors:** Tatsuo Yamamoto, Tomomi Takano, Wataru Higuchi, Yasuhisa Iwao, Olga Singur, Ivan Reva, Yuta Otsuka, Toru Nakayashiki, Hirotada Mori, Galina Reva, Vladimir Kuznetsov, Vladimir Potapov

This article described the ST239 variant in Vladivostok and the mobile element MES16K. The previous publication below by Chen et al. described a composite transposon named Tn6072:

Identification of a novel transposon (Tn6072) and a truncated staphylococcal cassette chromosome mec element in methicillin-resistant Staphylococcus aureus ST239.

Chen L, Mediavilla JR, Smyth DS, Chavda KD, Ionescu R, Roberts RB, Robinson DA, Kreiswirth BN.

Antimicrob Agents Chemother. 2010 Aug;54(8):3347-54.

A BLAST analysis for both mobile elements shows that EMS16K (30,834 bp) is 95.5% homologous to Tn6072 (29,438 bp). EMS16K included three insertions (1,332 bp, 51 bp, and 11 bp), compared to Tn6072. The ccrC-carrying unit of EMS16K is 99.9% homologous to that of Tn6072. In addition, the ST239 variants described in this article share the following characteristics with strain BK16704 described by Chen et al.: both are spa type 351 (t030), both possess an SCCmec element classified as 3A.1.4, and both are susceptible to trimethoprim. It is possible that the Vladivostok ST239 variant is related to the Romanian one described by Chen et al.

The above findings by Chen et al. should have been cited in the article and we are therefore issuing this Correction to acknowledge the work reported by Chen et al. and make readers aware of the similarities between the mobile elements in the two publications.

In addition, the authors would like to also acknowledge the following publications:

Staphylococcal cassette chromosome mec (SCCmec) typing of methicillin-resistant Staphylococcus aureus strains isolated in 11 Asian countries: a proposal for a new nomenclature for SCCmec elements.

Chongtrakool P, Ito T, Ma XX, Kondo Y, Trakulsomboon S, Tiensasitorn C, Jamklang M, Chavalit T, Song JH, Hiramatsu K.

Antimicrob Agents Chemother. 2006 Mar;50(3):1001-12.

Chongtrakool et al. described SCCmec elements very similar to those described in this article in the strain JCSC1716 from Saudi Arabia. Chongtrakool et al. described Type 3A.1.4 SCCmec by PCR analysis, while in the current study, we determined the entire sequence of SCCmecIII.1.1.4 of ST239/spa351(t030) strain 16K in Russia.

Molecular characterization and susceptibility of methicillin- resistant and methicillin-susceptible Staphylococcus aureus isolates from hospitals and the community in Vladivostok, Russia.

Baranovich T, Zaraket H, Shabana II, Nevzorova V, Turcutyuicov V, Suzuki H. Clin Microbiol Infect. 2010 Jun;16(6):575-82.

Baranovich et al. described ST238/spa351(t030) as one of the most frequent MRSA spatypes in Vladivostok. We would therefore like to correct the statement under the Discussion noting that the spa351 (t030) type has been found only in Europe. 

