# Supplementary figures and images for: Comparative Genomics and Drug Resistance of a Geographic Variant of ST239 Methicillin-Resistant Staphylococcus aureus Emerged in Russia
Source: PLoS One. 2012 Jan 19;7(1):e29187. doi: 10.1371/journal.pone.0029187 (PMC3261861; doi:10.1371/journal.pone.0029187)

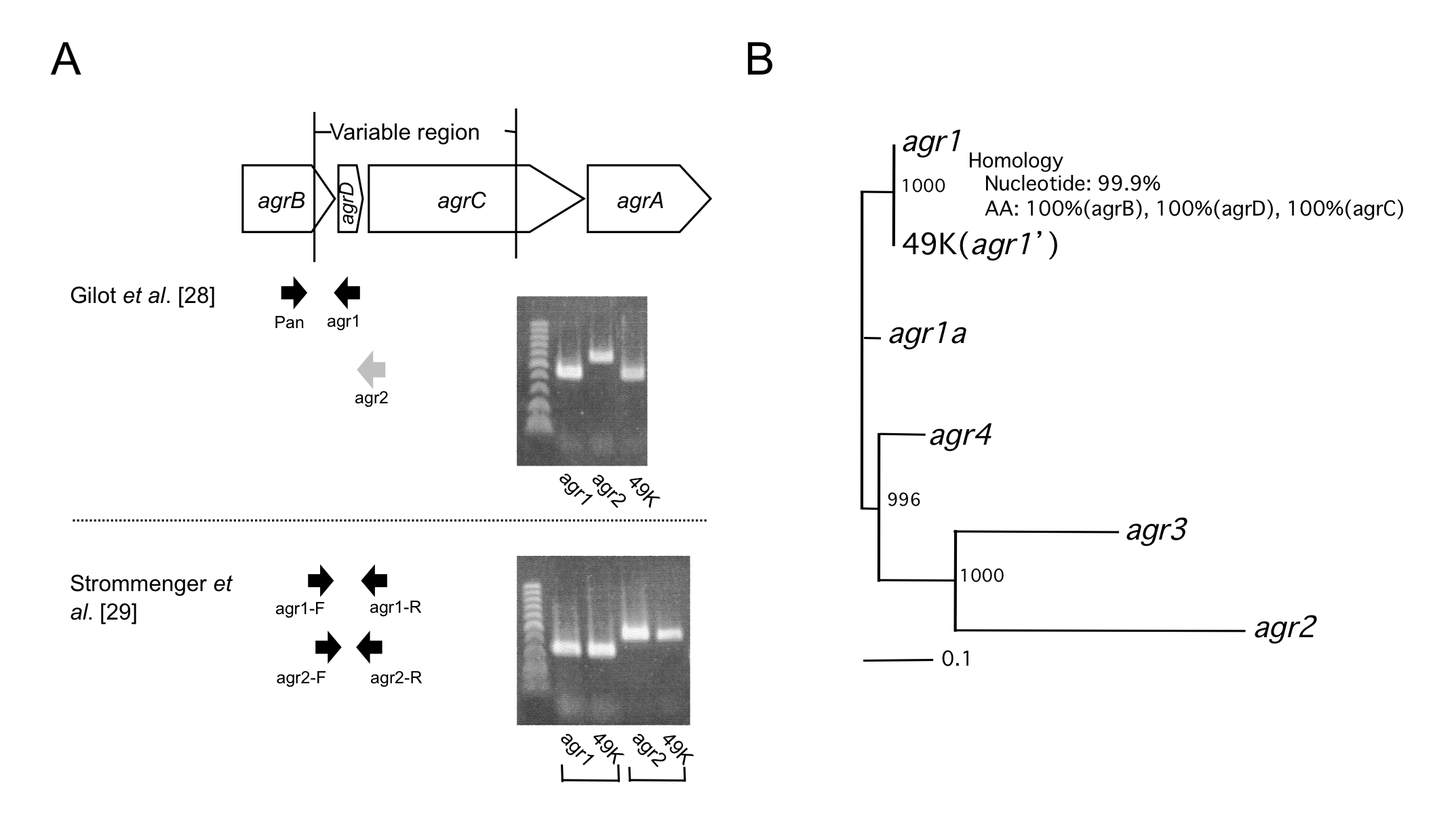

Supplement: Figure S1 — The agr subtyping for the Russian variant strain 49K. In A, PCR primers for agr typing are shown as an arrow. Strains: agr1, agr1 type strain; agr2, agr2 type strain; 49K, the Russian variant strain 49K. Strain 49K produced a product only for primer sets (Pan and agr1), and not for (Pan and agr2), assigning strain 49K as agr1. However, in the other PCR assay, strain 49K produced a product both for primer sets (agr1 forward and agr1 reverse) and for agr2 forward and agr2 reverse, resulting in no precise assignment. In B, the agr subtypes were examined by phylogenetic tree analysis, based on the nucleotide sequences of the agr variable region, as described previously [30]. GenBank accession number for the variable region sequence of strain 49K is AB592345. Based on the data, strain 49K was assigned as agr1′. (TIF) [file pone.0029187.s001.tif]

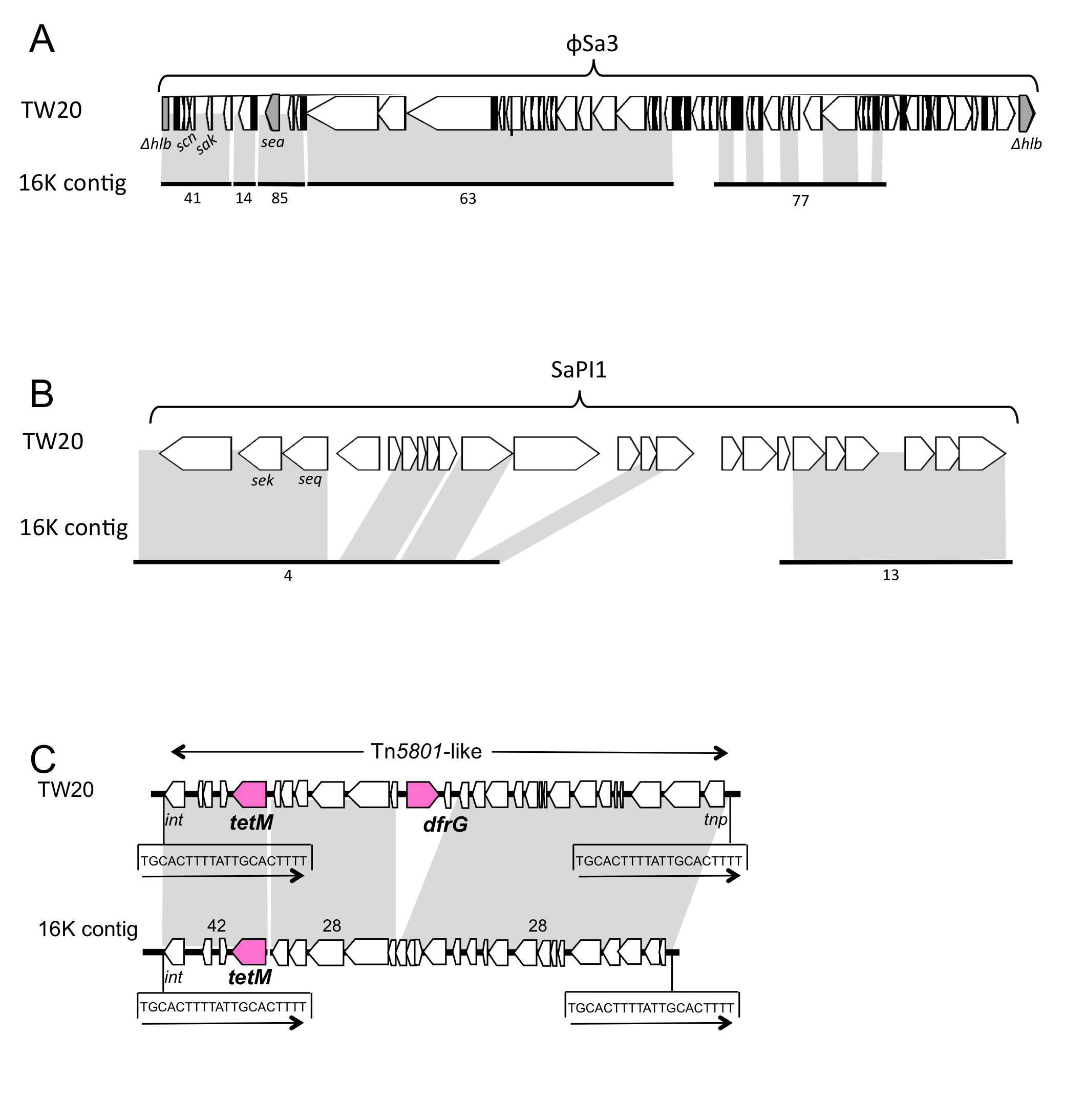

Supplement: Figure S2 — Deletions in drug resistance or virulence genetic traits in 16K, in comparison with TW20. The data of strain TW20 are from GenBank accession number FN433596. Homologous regions are shaded. In A, contigs 41, 14, 85, and 63 (and probably 77) of strain 16K constituted the bulk of the ΦSa3 phage sequence, showing the presence of the sea gene (encoding superantigen SEA, a virulence factor associated with severe invasive infections) in strain 16K; no right side sequence of ΦSa3 was present. In B, contigs 4 and 13 of strain 16K constituted the left and right side regions of the SaPI1 superantigen-associated pathogenicity island, showing the presence of the superantigen genes sek and seq in strain 16K. In C, contigs of 42 and 28 of strain 16K constituted the Tn5801-like sequence (including 20-bp direct repeat at both ends), but with deletion of the dfrG gene sequence (encoding trimethoprim resistance) in contig 28. Strain 16 was also negative for dfrG by PCR assay. (TIF) [file pone.0029187.s002.tif]
